# Miconazole protects blood vessels from MMP9-dependent rupture and hemorrhage

**DOI:** 10.1242/dmm.027268

**Published:** 2017-03-01

**Authors:** Ran Yang, Yunpei Zhang, Dandan Huang, Xiao Luo, Liangren Zhang, Xiaojun Zhu, Xiaolin Zhang, Zhenming Liu, Jing-Yan Han, Jing-Wei Xiong

**Affiliations:** 1 Beijing Key Laboratory of Cardiometabolic Molecular Medicine, Institute of Molecular Medicine, Peking University, Beijing 100871, China; 2 State Key Laboratory of Natural and Biomimetic Drugs, School of Pharmaceutical Sciences, Peking University, Beijing 100871, China; 3 Department of Integration of Chinese and Western Medicine, School of Basic Medical Sciences, Peking University, Beijing 100191, China; 4 AstraZeneca Asia and Emerging Market Innovative Medicine and Early Development, Shanghai 201203, China

**Keywords:** Chemical screen, Hemorrhage, Miconazole, Mmp9, Stroke, Zebrafish

## Abstract

Hemorrhagic stroke accounts for 10-15% of all strokes and is strongly associated with mortality and morbidity worldwide, but its prevention and therapeutic interventions remain a major challenge. Here, we report the identification of miconazole as a hemorrhagic suppressor by a small-molecule screen in zebrafish. We found that a hypomorphic mutant *fn40a*, one of several known *β-pix* mutant alleles in zebrafish, had the major symptoms of brain hemorrhage, vessel rupture and inflammation as those in hemorrhagic stroke patients. A small-molecule screen with mutant embryos identified the anti-fungal drug miconazole as a potent hemorrhagic suppressor. Miconazole inhibited both brain hemorrhages in zebrafish and mesenteric hemorrhages in rats by decreasing matrix metalloproteinase 9 (MMP9)-dependent vessel rupture. Mechanistically, miconazole downregulated the levels of pErk and Mmp9 to protect vascular integrity in *fn40a* mutants. Therefore, our findings demonstrate that miconazole protects blood vessels from hemorrhages by downregulating the pERK-MMP9 axis from zebrafish to mammals and shed light on the potential of phenotype-based screens in zebrafish for the discovery of new drug candidates and chemical probes for hemorrhagic stroke.

## Abstract (precis)

**Summary:** A phenotype-based chemical screen in zebrafish identifies miconazole as a novel hemorrhagic suppressor. Miconazole inhibits vessel rupture and hemorrhages by decreasing pErk and MMP9 in zebrafish and rats.

## INTRODUCTION

Cardiovascular diseases are the leading causes of mortality worldwide, and stroke ranks among the top three. In 2012, ∼6.7 million people died of stroke, accounting for ∼17% of total mortality from non-communicable diseases globally (WHO, 2015). Hemorrhagic stroke accounts for ∼10-15% of all strokes and is strongly associated with mortality and morbidity. Although the morbidity and mortality resulting from hemorrhagic stroke are high, we know less about its molecular pathology than we have learned from ischemic stroke. Current studies suggest that the inflammatory response is a major cellular event after hemorrhagic stroke; it is accompanied by neuronal death, leukocyte infiltration, and the activation of microglia/macrophages and astrocytes. Major inflammatory mediators include matrix metalloproteinases (MMPs), nuclear factor erythroid 2-related factor 2 (Nrf2), heme oxygenase (HO) and ferric irons (Wang, 2010). Various anti-inflammatory strategies in hemorrhagic stroke have been explored in both preclinical and clinical trials, such as microglia/macrophage inhibitory factor, MMP inhibitors, the Nrf2 inducer sulforaphane, HO inhibitors and deferoxamine for iron-mediated toxicity (Egashira et al., 2015; Wang, 2010). However, none of the above target-based treatments has been translated into the clinic so far.

The current failure to develop medical treatments, which primarily rely on target-based drugs, suggests that the essential therapeutic targets for this disease are not yet discovered, or alternatively, multiple genetic factors in parallel are involved in this complex disease. At the same time, phenotype-based drug discovery is gradually being recognized and actively pursued by both academic and industrial scientists using model organisms such as zebrafish (MacRae and Peterson, 2015). The zebrafish is a powerful model organism for both genetic and chemical screens, so establishing a suitable zebrafish model for hemorrhagic stroke would provide an alternative method in the search for medical treatment for this devastating disease.

Here, we present an ethylnitrosourea (ENU)-induced mutant *fn40a*, one of the known *β-pix* (*arhgef7b*) mutant alleles (Chen et al., 2001; Liu et al., 2007), as a hemorrhagic stroke model for chemical suppressor screens in zebrafish. By performing a small-molecule screen, we found that miconazole, a known anti-fungal drug, is a potent hemorrhagic suppressor. Further molecular and cellular analyses suggested that miconazole inhibited both brain hemorrhages in zebrafish and mesenteric hemorrhages in rats by decreasing MMP9-dependent vessel rupture. Therefore, our findings demonstrate the great potential of phenotype-based screens in zebrafish for the discovery of new drug candidates and chemical probes for hemorrhagic stroke.

## RESULTS

### The *fn40a* mutant is a suitable hemorrhagic stroke model for chemical suppressor screens in zebrafish

**Fig. 1.**
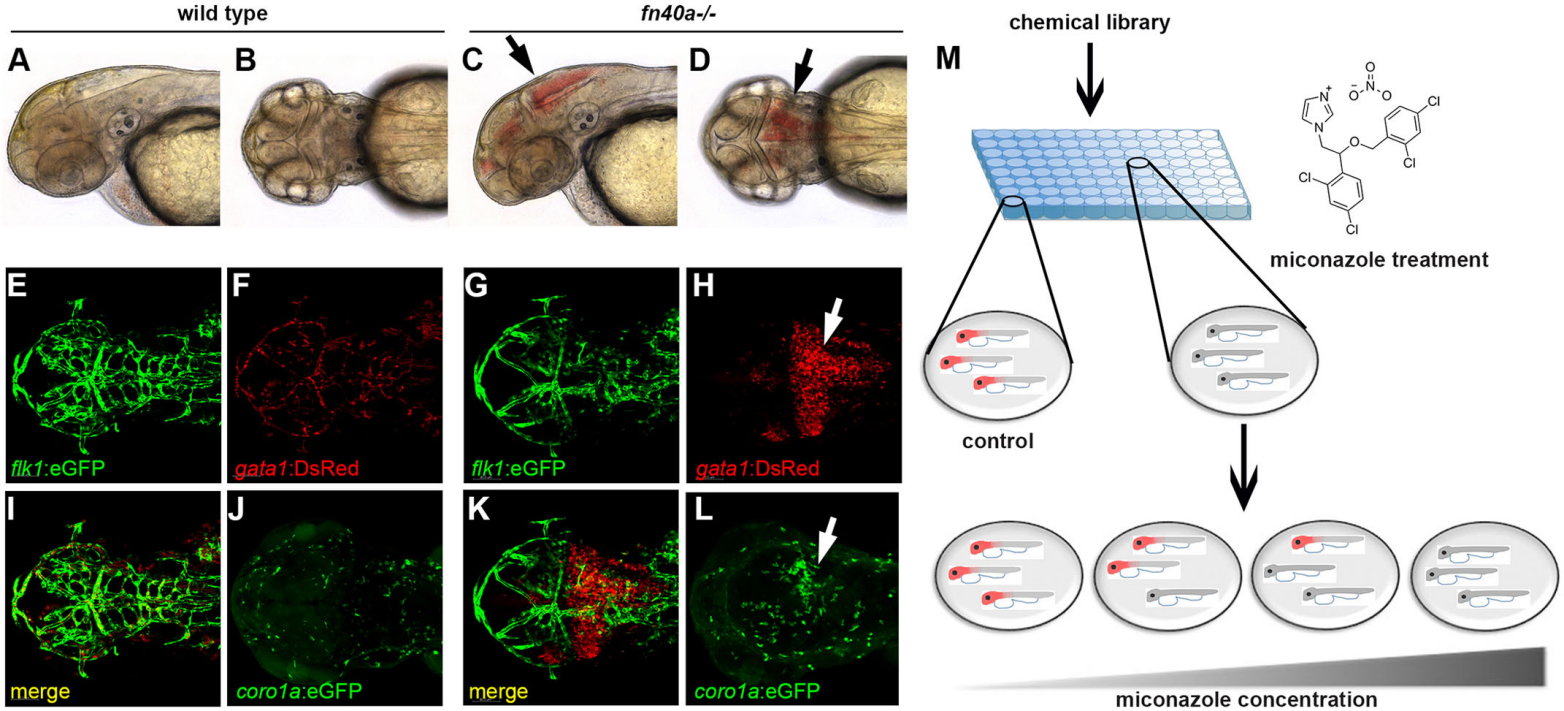
**The *fn40a* mutant is a hemorrhagic stroke model suitable for chemical suppressor screens in zebrafish.** (See also Figs S1,S2). (A-D) Live images of wild-type siblings (A,B) and homozygous *fn40a* mutants (C,D) at 2 dpf; note evident brain hemorrhages in the mutant (arrows). A,C: lateral view; B,D: dorsal view. (E-L) Live images of Tg(*flk1*:eGFP); Tg(*gata1*:DsRed) double transgenic embryos of wild-type siblings (E,F,I,J) and *fn40a* mutants (G,H,K,L) at 2 dpf. Note that *gata1^+^* erythrocytes leak from Tg(*flk1*:eGFP)-labeled vessels and form hematomas in the mutants (G-H,K; arrow) while erythrocytes remain within vessels in wild-type siblings (E,F,I). Tg(*coro1a*:eGFP)-labeled macrophages and neutrophils accumulate at the hemorrhagic site in this mutant (L, arrow) whereas leukocytes are evenly distributed in the brain of a wild-type sibling embryo (J) at 2 dpf (*n*>8). (M) Overview of the chemical screen with mutant embryos arrayed in a 96-well plate and treated with DMSO or compound, illustrating that miconazole suppresses brain hemorrhages in a dose-dependent manner compared with DMSO treatment. Red color indicates hemorrhagic mutants.

*fn40a* was identified as a hemorrhagic mutant from an ENU-induced mutagenesis screen of the zebrafish genome at Massachusetts General Hospital, Boston (Chen et al., 2001; Liu et al., 2007). This mutant failed to complement with *bubblehead^m292^*, a *β-pix* mutant, suggesting that *fn40a* is allelic to *m292*. However, no mutations were found in the coding region and splicing sites of *β-pix*, while *β-pix* mRNA decreased dramatically in homozygotic *fn40a* mutants (Liu et al., 2007), suggesting that mutations might occur in the promoter or enhancer region. While *m292* mutants are embryonic-lethal, we found that *fn40a* mutants are able to recover and survive to adulthood, confirming that *fn40a* is a hypomorphic allele to *β-pix* (Liu et al., 2007). This particular feature of viable homozygous *fn40a* adults, from which 100% mutant embryos can be obtained, was then exploited for a chemical suppressor screen as previously described for *gridlock* mutants in zebrafish (Peterson et al., 2004). Briefly, we inbred and collected the *fn40a* mutant embryos, and raised those with severe brain hemorrhage to the next generation. After two generations, the hemorrhage phenotype was severe and stable and the hemorrhage rate reached almost 100% (*n*>1000). Hemorrhagic sites occurred most frequently in the hindbrain and occasionally in the midbrain and forebrain of the mutants at 2 days post-fertilization (dpf) (Fig. 1C,D; *n*>100), which is similar to another *β-pix* allele, *m292* (Liu et al., 2007, 2012) and redhead mutant, *pak2a* (*mi149*) (Buchner et al., 2007). Since the hemorrhages of homozygous mutants dissolved gradually and disappeared around ∼4 dpf, they could survive to adulthood with normal body growth and fertility. By using *flk1*:eGFP and *gata1*:DsRed double transgenic zebrafish, we found that *gata1*:DsRed-labeled erythrocytes flowed completely inside well-patterned brain vessels labeled by *flk1*:eGFP in wild-type sibling embryos (Fig. 1E,F,I; Movie 1). In contrast, erythrocytes accumulated in the intracerebral region of *fn40a* mutants (Fig. 1H,K), accompanied by disruption of the central arteries (CtAs) between the basilar artery (BA) and the primordial hindbrain channel (PHBC) in the hindbrain (Fig. 1G,K; Movie 2). The recruitment of *coro1a*:eGFP-labeled inflammatory cells to the hemorrhagic region in mutants (Fig. 1L) compared with the evenly distributed inflammatory cells in wild-type siblings (Fig. 1J). In addition, it has been reported that *β-pix* interacts with *rap1b* and *ccm1*, in which *ccm1* is one of the mutated genes causing cerebral cavernous malformations (Gore et al., 2008), thus establishing a potential link between this mutant and intracranial hemorrhages in humans. These data suggest that, like intracerebral hemorrhagic models in rodents (Wang, 2010), the *fn40a* mutant mimics the phenotypes of hemorrhagic stroke, such as brain-vessel rupture, intracerebral hemorrhage and inflammation; and the transient brain hemorrhages of the mutants can be exploited for a chemical suppressor screen in zebrafish.

Using an in-house chemical library with diversified chemical structures of 923 drug-like compounds, we performed a hemorrhagic suppressor screen. The outline of this screen includes three major steps (Fig. 1M). During the first step (primary screen), 5 *fn40a* mutant embryos were arrayed in each well of 96-well plates, and were then subjected to administration of 10 µmol/l of small molecules from 6 to 24 hpf. At 24 hpf, the compounds were washed away, replaced, and embryos were incubated with fresh egg water until the desired stages. Each compound was replicated in three wells. The hemorrhagic suppression efficiency was scored based on the hemorrhage rate for each compound. The second step was to validate the efficacy of candidate compounds from the primary screen by using an average of 200 mutant embryos. In the final step of validation, we tested the dose-dependent efficacy and toxicity of each candidate compound with a series of concentrations and more than 70 mutant embryos per condition. Through this screen, we isolated 7 candidate hemorrhagic suppressors (chemical structures are shown in Fig. S1) and their hemorrhagic suppression efficacy is shown by the hemorrhage rate (Table S1). Miconazole nitrate stood out of the seven candidates because of its great efficacy on hemorrhagic suppression and least toxicity to embryos. Importantly, miconazole effectively suppressed brain hemorrhages in a dose-dependent manner (Fig. S2N). By applying 5 μmol/l miconazole to *fn40a* mutant embryos from 6 to 24 hpf, we found that it suppressed ∼70% of brain hemorrhages compared with levels in DMSO-treated embryos (Fig. S2N). In addition, application of miconazole from 6 to 24 hpf had more of an effect on suppression of brain hemorrhage than did treatment from either 24 to 36 hpf or 24 to 48 hpf, and treatment from 36 to 48 hpf had no inhibition effects (Fig. S2O). This suggests that miconazole plays a protective role in generating mature cerebral vessels before their ruptures and hemorrhages occur in *fn40a* mutants around 40 hpf, and/or that miconazole might take longer to reach its target and have any action *in vivo*. Taken together, we have identified a suitable hemorrhagic stroke model in zebrafish that could be used to identify the anti-fungal drug miconazole as a novel, potent hemorrhagic suppressor of *fn40a* mutants.

### Miconazole suppresses intracerebral hemorrhages in *fn40a* mutants

**Fig. 2.**
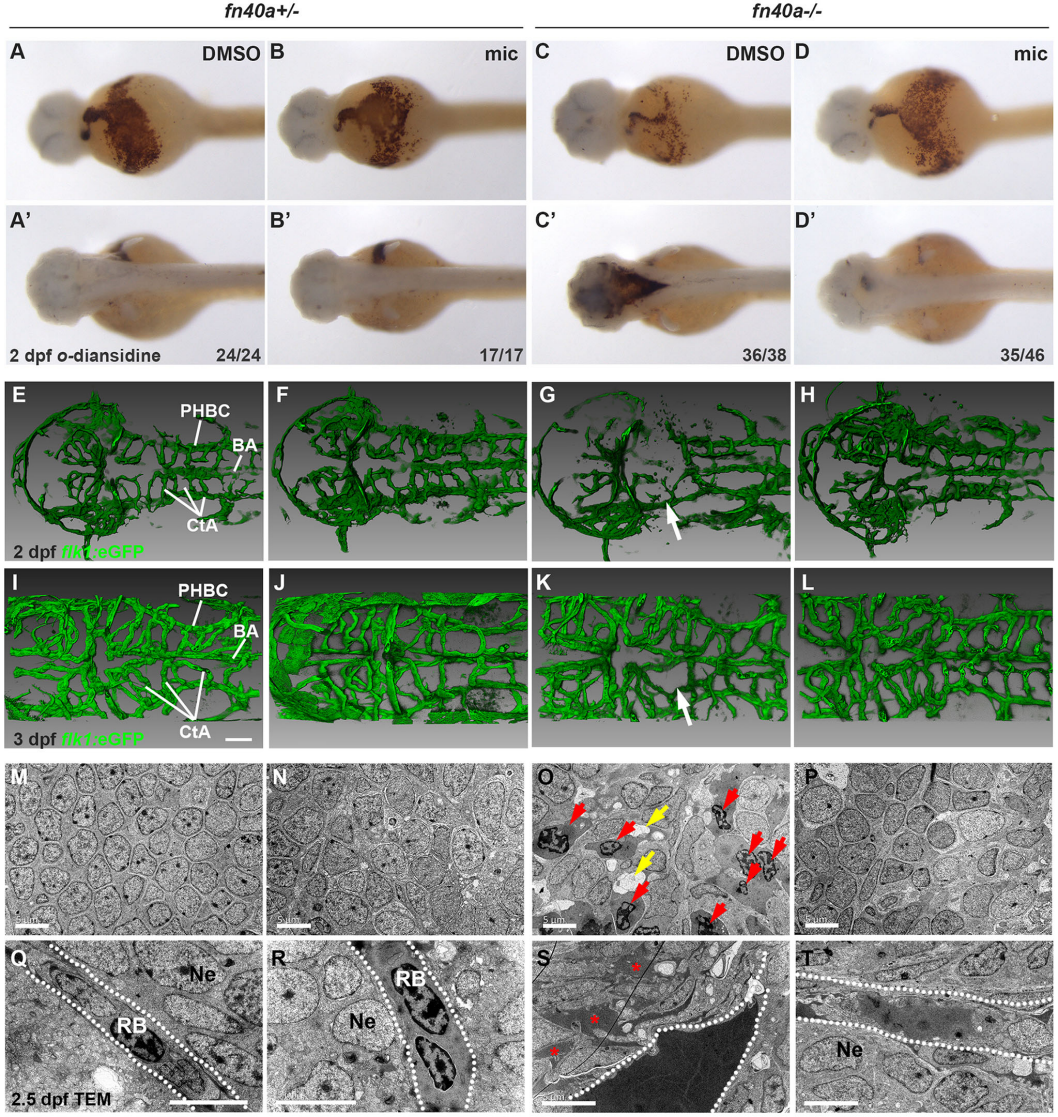
**Miconazole suppresses brain hemorrhages in *fn40a*^−/−^ mutants.** (See also Figs S2-S4 and Table S1). (A-D′) *O*-dianisidine staining for erythrocytes showing hemorrhages in the dorsal part of the brain (C′) that were suppressed by miconazole (mic) treatment (D′) in homozygous *fn40a* mutants whereas no brain hemorrhages occur in heterozygous *fn40a* mutants in the absence or presence of miconazole (mic) (A-B′) at 2 dpf. A-D, ventral view; A′-D′, dorsal view. The numbers on the lower right show the number of hemorrhagic (C,C′) and non-hemorrhagic (A-B′,D,D′) embryos out of the total embryos scored. (E-L) CtA between BA and PHBC labeled in Tg(*flk1*:eGFP) zebrafish are disrupted (G,K, arrows) in homozygous mutants. This is rescued by mic treatment (H,L) in homozygous mutants compared with heterozygous mutants with either DMSO or mic treatment (E-F,I-J). Images are representative of more than 10 embryos per group (*n*>10). E-H, 2 dpf; I-L, 3 dpf. Scale bars: 50 µm. (M-T) Transmission electron microscopy shows the invasion of blood cells (red arrows) and disrupted neurons (O) and the leakage of high-density blood plasma (red asterisks) into the surrounding brain tissue (S) in *fn40a* homozygous mutants. This is rescued by mic treatment (P,T) compared with heterozygous mutants with or without mic treatment (M-N, Q-R) at 2.5 dpf. Images are representative of more than 8 embryos per group (*n*>8). In Q-T, dashed lines mark blood vessel border; red arrows indicate invaded blood cells; yellow arrows indicate vacuoles after cell death. BA, basilar artery; CtA, central artery; PHBC, primordial hindbrain channel; RB, red blood cell; Ne, neuron. Scale bars: 5 μm.

**Fig. 3.**
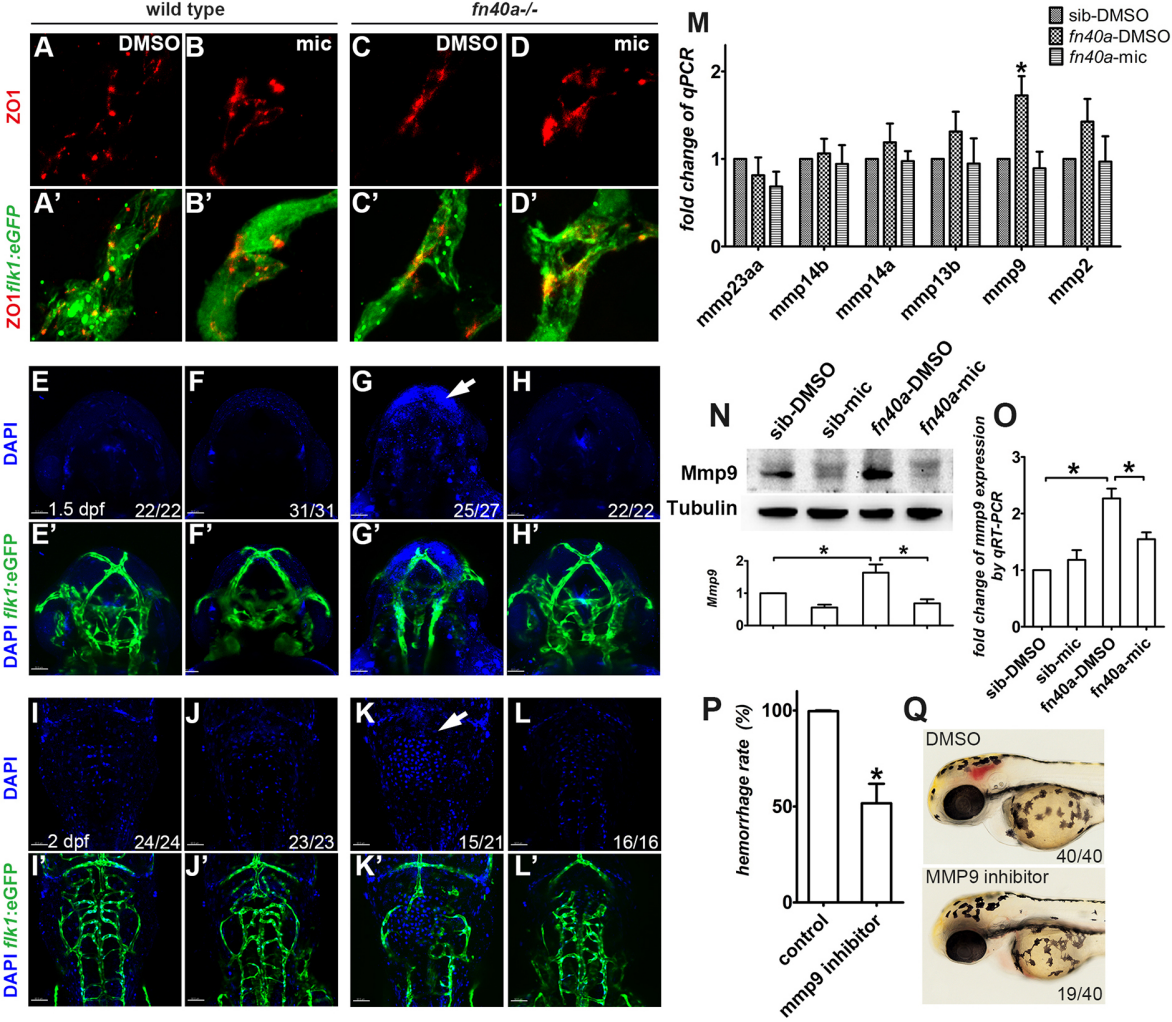
**Miconazole suppresses brain hemorrhages by preventing Mmp9-mediated vessel disruption in *fn40a*^−/−^ mutants.** (See also Fig. S5). (A-D′) Fluorescence immunohistochemistry reveals comparable expression of the tight junction protein ZO1 in Tg(*flk1*:eGFP)-labeled brain vessels with or without miconazole (mic) treatment in wild-type siblings (A-B′) and *fn40a* mutants (C-D′) at 2 dpf. Images are representative of more than 15 embryos per group (*n*>15). (E-L′) Microinjection of DAPI into the sinus venous revealed that neurons are stained by leaked DAPI before (G,G′, arrow) and after (K,K′, arrow) evident hemorrhages, suggesting that vascular permeability increases in homozygous *fn40a* mutants. This was rescued by mic treatment (H,H′,L,L′), compared with much less leakage of DAPI in siblings with or without mic treatment (E-F′,I-J′). E-H′ are embryos at 1.5 dpf, I-L′ are embryos at 2 dpf. The numbers on the lower right show the number of phenotypical embryos out of the total embryos scored. Scale bars: 100 µm. (M) Quantitative real-time PCR identified that *mmp9*, but not other MMP genes, is upregulated in homozygous *fn40a* mutants and that was suppressed to the normal level by mic treatment at 2 dpf. *P<0.05 by two-way ANOVA with Bonferroni post-tests, compared with sib-DMSO group for each gene; means±s.e.m. (N,O) Western blot (N) and qRT-PCR (O) confirmed that both MMP9 protein and *mmp9* mRNA increases in the mutants with DMSO control treatment and this is normalized by mic treatment, compared with wild-type siblings with or without mic treatment at 2 dpf. *P<0.05 by one-way ANOVA with Bonferroni's multiple comparison test, means±s.e.m. Total RNA was extracted from whole embryos and protein from whole embryos without yolk. (P,Q) MMP9 inhibitor I suppresses brain hemorrhage rate to 50% in homozygous mutants (P), in which brain hemorrhage was scored in live mutant embryos treated with DMSO or MMP9 inhibitor (Q). *P<0.05 by Student's *t*-test, compared with controls; means±s.e.m.

*O*-dianisidine staining of live embryos allowed us to compare the amounts of erythrocytes in heterozygous and homozygous *fn40a* mutants in the presence of PTU (0.003%) (Fig. 2A,A′,C,C′). While erythrocytes were found in the heart, common cardinal vein, dorsal aorta and other major vessels in heterozygous siblings treated with DMSO or miconazole at 2 dpf (Fig. 2A-B′), brain hemorrhages were evident in homozygous mutants (Fig. 2C′) and were suppressed by miconazole treatment (Fig. 2D′). We found that hemorrhage rate in normal pigmented mutant embryos was similar to that in PTU-treated mutant embryos, which were also suppressed by miconazole (Fig. 2A-D, A′-D′; Fig. S2A-H). To quantify the level of hemorrhage, we applied Tg(*flk1*:eGFP) to label vascular endothelial cells and Tg(*gata1*:DsRed) to label erythrocytes. Consistent with the data from *O*-dianisidine staining, we found that brain hemorrhage was evident in homozygous mutants compared with heterozygous siblings in which miconazole inhibited hemorrhage (Fig. S2I-M). By testing five known analogues of miconazole, we found that sulconazole and tioconazole efficiently inhibited brain hemorrhage while voriconazole, fluconazole or ketoconazole had little effect (Fig. S3; *n*>50), suggesting that the imidazole group is essential for the activity of hemorrhagic suppression of miconazole. To address whether vessel patterning defects occur in homozygous mutants, we bred *flk1*:eGFP transgenic zebrafish into the *fn40a* mutant background. The *flk1*:eGFP-labeled cerebral vessels were well patterned and symmetrical in heterozygous siblings treated with DMSO or miconazole at 2 dpf and 3 dpf (Fig. 2E,F,I,J). By contrast, some of the CtAs were disrupted in homozygous *fn40a* mutants treated with DMSO at 2 dpf (Fig. 1G) and 3 dpf (Fig. 1K), consistent with the previous report on *bubblehead^m292^* (Liu et al., 2007). Remarkably, miconazole treatment protected the CtAs from rupture and hemorrhage (Fig. 2D′,H,L).

To gain insights into the hemorrhage processes, we first set up time-lapse 3-dimensional imaging to monitor the development of brain vessels in live embryos using a LSM700 confocal microscopy. The *fli1a:nuc*-eGFP transgenic reporter was used to label the nuclei of both endothelial cells and erythroid cells, allowing us to easily identify proliferation and migration of these cells during development. In *fn40a* mutants, cerebral vessels were well-patterned and grew normally up to 1.5 dpf, around which time blood cells frequently leaked out from the central arteries to form hematomas, resulting in further inflammation and disruption of the surrounding vessels (Movie 4; *n*>5) compared with heterozygous siblings (Movie 3; *n*>5). These data suggest that cranial vascular patterning is relatively normal but those vessels are immature and fragile in the *fn40a* mutant.

To visualize the subcellular structures of cerebral vessels and surrounding cells, we used transmission electron microscopy (TEM) and found that blood cells were normally located inside blood vessels and did not infiltrate into surrounding neurons in heterozygous mutants (DMSO treatment) (Fig. 2M,Q), which were not affected by miconazole treatment (Fig. 2N,R). In contrast, blood cells invaded neuronal tissues (Fig. 2O) in mutants and blood vessels were disrupted and a large amount of electron-dense blood plasma diffused outward and interrupted the interactions of surrounding cells in mutants (Fig. 2S). These abnormalities were rescued by miconazole, which restored the restriction of blood cells and plasma within blood vessels in mutants (Fig. 2P,T). Similarly, microvessels were also disrupted in mutants and these defects were rescued by miconazole treatment compared with heterozygous siblings with or without miconazole treatment (Fig. S4). Therefore, our data reveal the critical role of miconazole in protecting cerebral vessels from rupture and hemorrhage in *fn40a* mutants.

### Miconazole improves vessel integrity by decreasing Mmp9 in *fn40a* mutants

The above data suggested that the integrity of brain vessels was compromised in *fn40a* mutants. We then tested whether vascular permeability was affected in this mutant by examining the leakage of DAPI injected through the sinus venous of wild-type embryos (Fig. 3E-F′,I-J′) and *fn40a* mutants (Fig. 3G-H′,K-L′) at 36 hpf (Fig. 3E-H′; before evident brain hemorrhage) and 48 hpf (Fig. 3I-L′; after initiation of brain hemorrhage). As expected, we found a little DAPI leakage from brain vessels in wild-type siblings at 36 hpf (Fig. 3E,E′) and 48 hpf (Fig. 3I,I′), and this was not affected by miconazole treatment (Fig. 3F,F′,J,J′). In contrast, we found a large amount of DAPI leaked from blood vessels, resulting in DAPI-stained neurons in *fn40a* mutants (DMSO control) before (36 hpf) or after (48 hpf) brain hemorrhage occurred, respectively (Fig. 3G,G′,K,K′), and this was rescued by miconazole (Fig. 3H,H′,L,L′). These data strongly support the notion that brain vessels are compromised, with higher permeability in *fn40a* mutants.

In mammals, the neurovascular unit (NVU) maintains cerebral homeostasis (Posada-Duque et al., 2014). NVU includes the cellular components of vascular endothelia, neurons and non-neuronal cells (microglia, astrocytes, and pericytes), as well as the molecular components of intercellular junction proteins such as tight junctions and extracellular proteins like MMPs (Xing et al., 2012). Since we found minimal changes in the structure and number of neurons and endothelial cells in the *fn40a* mutant by fluorescent transgenic reporters, we wondered whether the molecular components were affected. Fluorescence immunohistochemistry showed comparable zonula occludens-1 (ZO1) expression in wild-type siblings and mutants, and this was not affected by miconazole (Fig. 3A-D′), suggesting that tight junctions were probably less affected at this level. Using TEM analysis, we found that cerebral endothelial cells were disrupted in mutants, and these defects were rescued by miconazole treatment (Fig. S4). On the other hand, we did not find any blood-brain barrier structure outside the endothelium, as observed in mammals by TEM, and blood cells and plasma flowed exclusively in blood vessels with no or little leakage of DAPI in 2.5 dpf embryos. These data confirm that miconazole protects these immature cerebral vessels at this early embryonic stage in zebrafish.

Previous research in rodents has demonstrated that MMPs are essential components for regulating vascular integrity (Xing et al., 2012). The increasing level of MMPs enhances proteolysis in the extracellular matrix (ECM) and facilitates cell proliferation and migration during development and metastasis. More importantly, MMPs, which are well-known inflammatory mediators, play an important role in brain injury (del Zoppo, 2010; Xing et al., 2012). In the zebrafish genome, only six MMP members are well annotated: *mmp2*, *mmp9*, *mmp13b*, *mmp14a*, *mmp14b* and *mmp23aa*. We next tested whether these MMPs were affected in *fn40a* mutants, and if so, whether they were responsive to miconazole treatment. Quantitative real-time PCR revealed that *mmp9*, but not the other MMP genes, was significantly upregulated in *fn40a* mutants (DMSO control), and this was suppressed by miconazole, compared with wild-type siblings (DMSO control) at 2 dpf (Fig. 3M). Additional analyses showed that both Mmp9 protein (Fig. 3N) and *mmp9* mRNA (Fig. 3O) were upregulated in *fn40a* mutants, and these were effectively rescued by miconazole. Miconazole had no effect on *mmp9* mRNA but decreased Mmp9 protein level in wild-type siblings (Fig. 3N), suggesting that it might also regulate Mmp9 protein stability. In addition, whole-mount *in situ* hybridization revealed that *mmp9* RNA was enriched in the brain at 1.5 and 2 dpf, and it increased in homozygous mutants, which was suppressed by miconazole (Fig. S5A-H). Importantly, elevated Mmp9 proteins were associated with brain vessels of homozygous mutants compared with heterozygous mutants in the absence or presence of miconazole; and like *mmp9* mRNA, Mmp9 protein was also suppressed by miconazole in brain vessels of the homozygous mutant (Fig. S5I-Q).

MMP9 is highly conserved among species from zebrafish, mouse, rat, and rabbit to human (Fig. S6). Consistently, MMP9 inhibitor I (3 µmol/l) effectively decreased the brain hemorrhage rate from 100% (in mutants with DMSO) to ∼50% (in mutants with MMP9 inhibitor I) (Fig. 3P,Q). Mutants treated with MMP9 inhibitor I had no or little hemorrhage compared with those in mutants by DMSO (Fig. 3Q). In addition, two other MMP9 inhibitors (II and V) had a similar effect on inhibiting hemorrhage in a dose-dependent manner (Fig. S7E-F). MMP9 inhibitor II at 4 µmol/l had very low solubility in E3 medium, which might be the cause of its reduced efficiency in inhibiting hemorrhage (Fig. S7E). In addition, MMP9 zymography assay confirmed that MMP9 inhibitor I, MMP9 inhibitor II and miconazole decreased Mmp9 activity in homozygous *fn40a* mutants compared with DMSO-treated mutants (Fig. S7G). Taken together, our data suggest that the increased permeability of brain vessels and the resultant hemorrhages are at least partly due to elevated Mmp9 in *fn40a* mutants, and miconazole suppresses the hemorrhages by decreasing Mmp9 expression.

### Miconazole has a conserved function in protecting mesenteric vessels against MMP9-dependent hemorrhagic rupture in rats

**Fig. 4.**
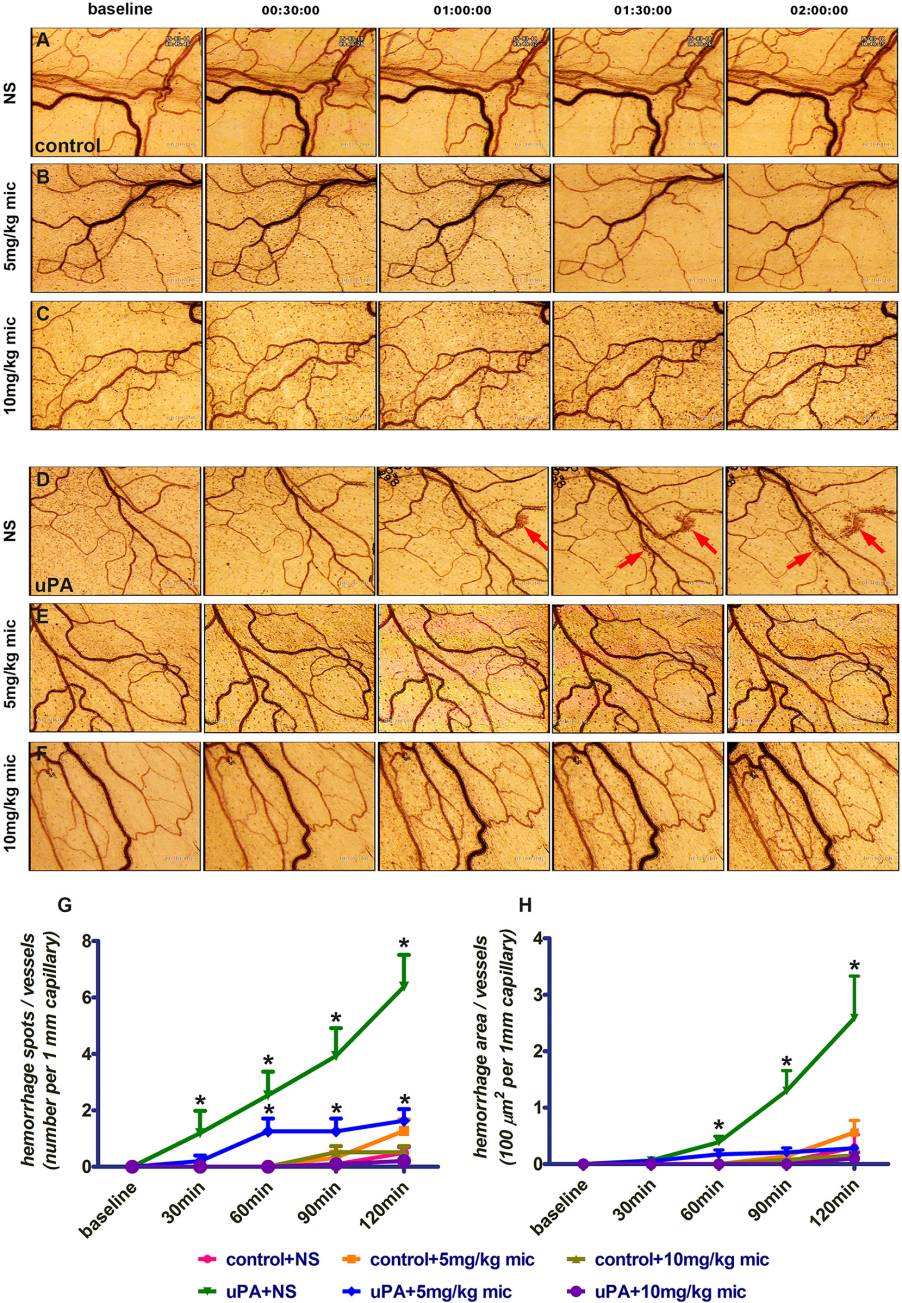
**Miconazole suppresses uPA-induced mesenteric hemorrhage in rats.** (A-C) Neither physiological saline (NS) nor miconazole (mic) at 5 or 10 mg/kg causes mesenteric hemorrhages 2 h after treatment in the control animals. (D-F) uPA treatment induces mesenteric hemorrhages from 1 to 2 h post-treatment (arrows) in the presence of NS (D). This is suppressed by pre-treatment with miconazole at either 5 or 10 mg/kg (E,F). (G,H) Both the number of hemorrhagic spots (G) and the hemorrhagic area (H) induced by uPA treatment are significantly decreased by pre-treatment with 5 or 10 mg/kg miconazole compared with NS control without uPA treatment. *P<0.05 by two-way ANOVA with Bonferroni post-test compared with baseline (*n*>5); means±s.e.m.

Having found that miconazole is an effective hemorrhagic suppressor in zebrafish, we then tested whether it also played a similar role in mammals. Extending this study to mammals was critical for both therapeutic utility and mechanistic studies. To achieve this goal, we established a mesenteric hemorrhagic model in rats by using the urokinase-type plasminogen activator (uPA)-dependent activation of MMP9, degradation of the ECM, and resultant mesenteric hemorrhages. Compared with control treatment with physiological saline (Movies 5 and 6), we found that miconazole (5 or 10 mg/kg by intravenous injection) did not cause mesenteric hemorrhages (Fig. 4A-C), suggesting that miconazole had little toxicity at the concentrations we used. By applying uPA (30,000 IU/kg) in rats, we started to detect mesenteric hemorrhages at ∼60 min, and more hemorrhages from 90 to 120 min after uPA induction (Fig. 4D; Movies 7 and 8). In contrast, pre-treatment with miconazole (5 or 10 mg/kg) 30 min before uPA injection prevented mesenteric hemorrhages (Fig. 4E,F) in terms of both the number of hemorrhagic spots and the hemorrhagic area (Fig. 4G,H). Together with its role in the maturation of developing cerebral vessels in zebrafish (Figs 2 and 3; Fig. S2O), these pharmacological data suggest that miconazole plays a conserved role in protecting vessels against MMP9-dependent rupture in rats. Therefore, identification of chemical suppressors such as miconazole for developing cerebral vessel ruptures has important implications in decreasing adult pathological phenotypes including vessel ruptures and hemorrhages.

**Fig. 5.**
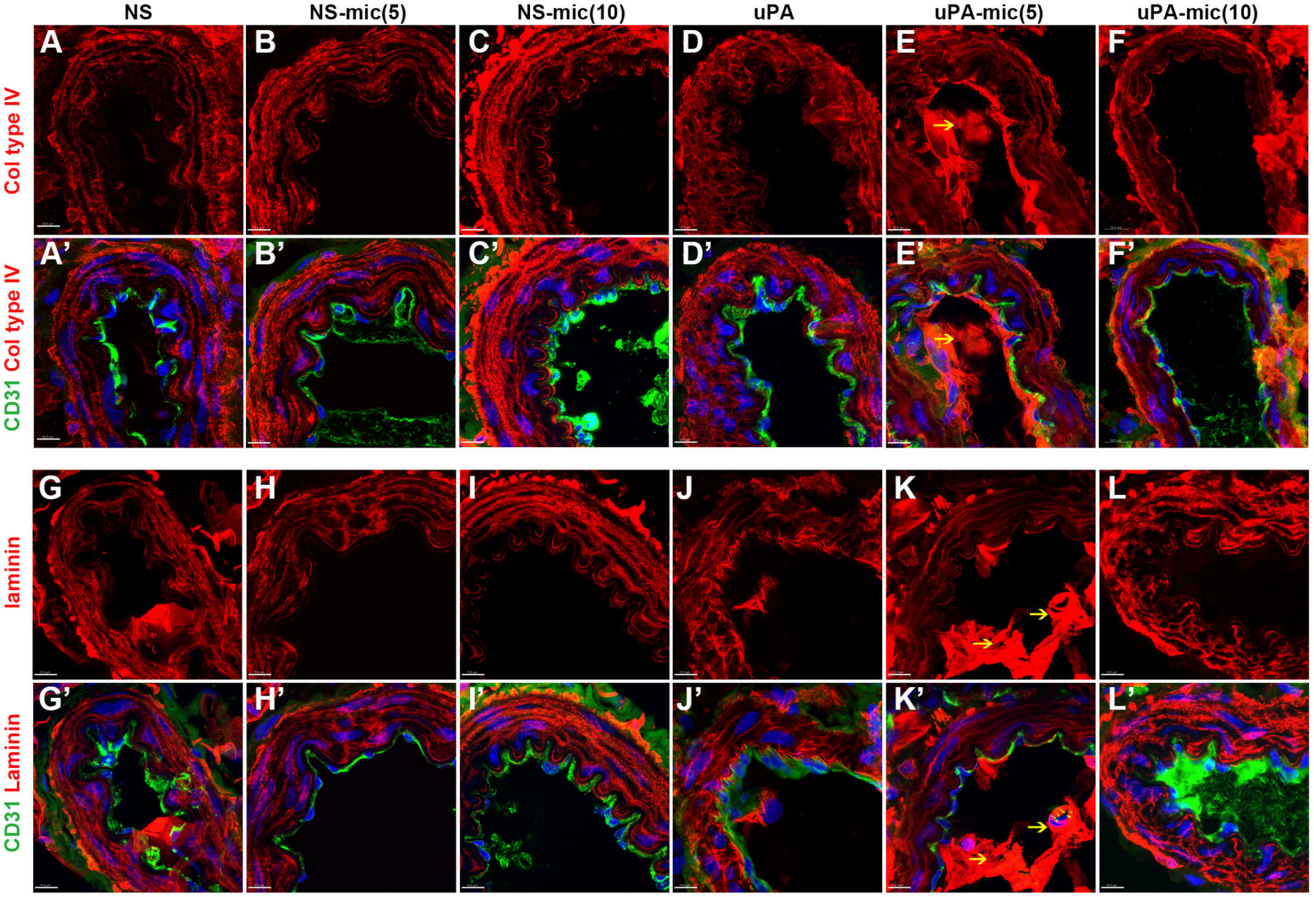
**Miconazole ameliorates uPA-induced degradation of the extracellular matrix (ECM) of mesenteric vessels in rats.** (A-F′) Fluorescence immunohistochemistry shows that the fiber-like ECM structure, stained for collagen (Col) type IV (red), is disrupted by uPA treatment (D,D′). This was partly rescued by either 5 or 10 mg/kg miconazole (mic) treatment (E-F′), compared with NS or NS-mic without uPA treatment (A-C′). (G-L′) Fluorescence immunohistochemistry shows that the fiber-like ECM structure, stained for laminin (anti-Lama1, red), is disrupted by uPA treatment (J,J′). This was partly rescued by either 5 or 10 mg/kg mic (K-L′), compared with NS or NS-mic without uPA treatment (G-I′). CD31 (green) was used to label the endothelial layer of the mesenteric vessels and DAPI (blue) was used to stain nuclei. The number of animals used in each group is more than 5 (*n*>5). Yellow arrows indicate non-specific staining in E,E′ and K,K′. Scale bars: 100 µm.

**Fig. 6.**
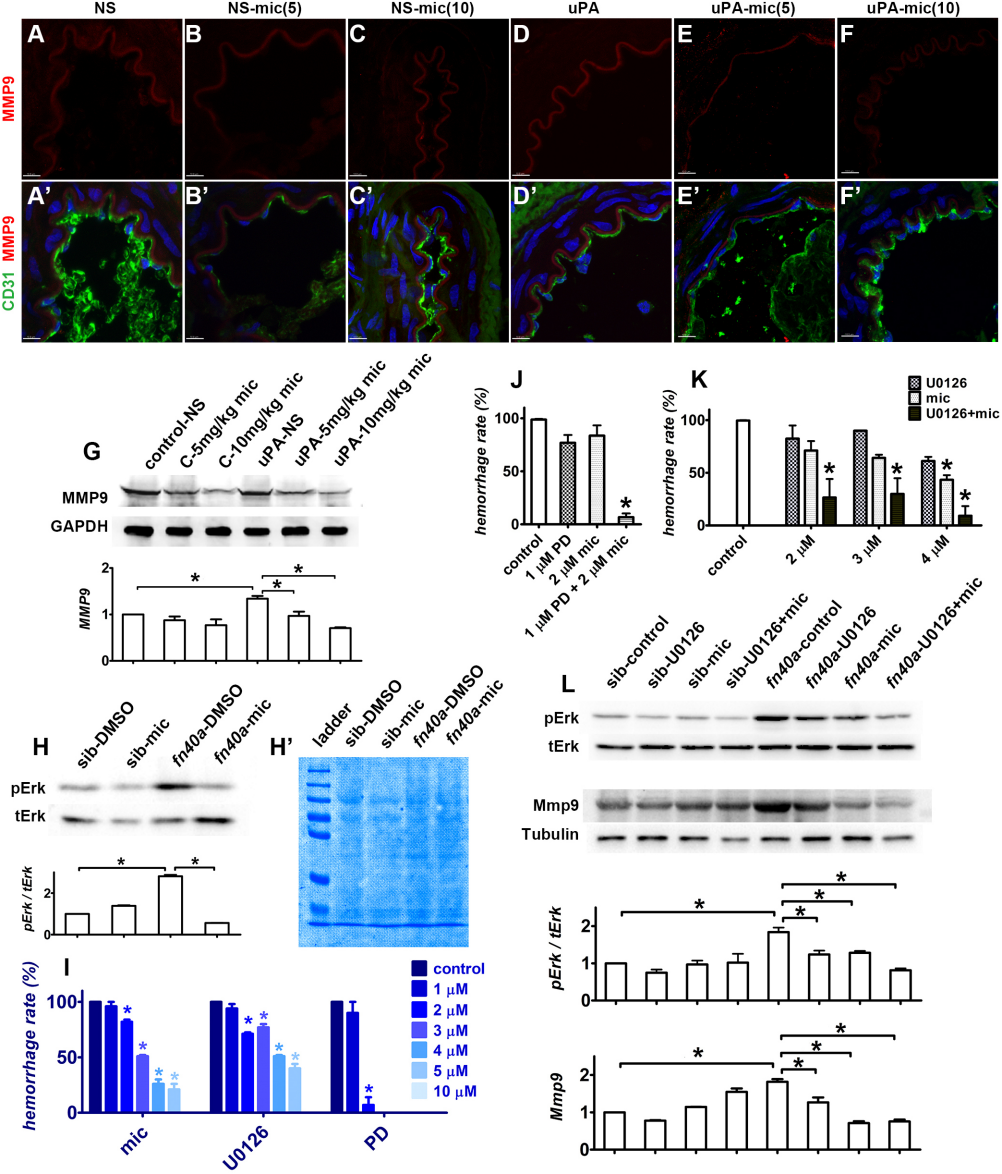
**Miconazole suppresses hemorrhages via the MEK-ERK-MMP9 pathway.** (See also Fig. S8). (A-F′) Fluorescence immunohistochemistry of rat mesenteric vessels showed that MMP9 (red) is induced in the ECM by uPA treatment (D,D′). This was rescued by either 5 or 10 mg/kg miconazole (mic) (E-F′), compared with those without uPA treatment (A-C′). CD31 (green) was used to label the endothelial layer of mesenteric vessels and DAPI (blue) was used to stain nuclei. The number of animals used in each group is more than 5 (*n*>5). Scale bars: 100 µm. (G) Western blots confirm that the uPA-induced expression of MMP9 is rescued by pre-treatment with mic. (H,H′) pErk increases in homozygous zebrafish *fn40a* mutants (DMSO control) and this was reversed by mic (5 µmol/l), compared with wild-type siblings with DMSO or mic at 24 hpf (H). Coomassie blue staining shows protein loading (H′). (I-L) As for mic, both MEK inhibitors U0126 and PD0325901 suppress the brain hemorrhages in homozygous *fn40a* mutants in a dose-dependent manner (I). Mic and PD0325901 (J) or U0126 (K) synergistically suppress brain hemorrhages in this mutant. Miconazole (3 µmol/l) and U0126 (3 µmol/l) synergistically suppress pErk, as well as Mmp9 in *fn40a* mutants at 24 hpf, compared with wild-type siblings with DMSO or miconazole treatment (L). *P<0.05 by one-way ANOVA with Bonferroni's multiple comparison test (G,H,J,L), or two-way ANOVA with Bonferroni post-tests compared with control (I,K); means±s.e.m.; *n*>50.

Previous studies have demonstrated that uPA cleaves plasminogen to generate the active proteinase plasmin; and plasmin can reciprocally activate pro-uPA. Plasmin cleaves and activates pro-matrix metalloproteases (pro-MMPs) including pro-MMP9 to activated MMPs; and both plasmin and MMPs break down many components of the ECM (Smith and Marshall, 2010). In the absence of uPA, we found a well-organized ECM, labeled by either collagen type IV or laminin (Lama1) in mesenteric microvessels treated with physiological saline (Fig. 5A,A′,G,G′), 5 mg/kg miconazole (Fig. 5B,B′,H,H′) or 10 mg/kg miconazole (Fig. 5C,C′,I,I′). CD31 labeled the vascular endothelium and DAPI was used to stain the nuclei. In the presence of uPA, the fibrillary ECM of mesenteric vessels was partly broken down and became less organized on transverse sections (Fig. 5D,D′,J,J′), and this was rescued by pre-treatment with miconazole at either 5 or 10 mg/kg (Fig. 5E-F′,K-L′). In addition, the tight junction proteins ZO1 and Claudin 5 decreased in the uPA-induced hemorrhagic model, and this was partially rescued by pre-treatment with miconazole (data not shown). As expected, MMP9 was upregulated in the ECM close to the endothelium in our model system, and this was effectively suppressed by pre-treatment with miconazole (Fig. 6A-F′). Western blot analysis further substantiated this conclusion (Fig. 6G). Our data has confirmed previous studies that uPA-induced MMP9 disrupts the ECM and tight junctions, establishing a suitable model of mesenteric hemorrhage, and, as in zebrafish, miconazole effectively suppresses MMP9-dependent vessel rupture and hemorrhage, highlighting the idea that miconazole acts on highly conserved protein components or signaling pathways.

### Miconazole suppresses brain hemorrhage by decreasing Erk1/2-Mmp9 signaling

Through a quantitative mass spectrum analysis of whole embryos, we found that β-Pix and five Pak-family members were downregulated in *fn40a* mutants but these changes were not rescued by miconazole treatment, compared with sibling heterozygotes (data not shown), suggesting that miconazole functioned downstream to the Pix/Pak pathway. In addition, previous studies have revealed a strong correlation between extracellular signal-regulated kinase 1 and 2 (ERK1/2) and MMP9 at both the transcriptional and post-transcriptional levels (Garavello et al., 2010). MMP9 is inducible and its expression is regulated by many transcriptional factors including activating protein 1 and 2 (AP-1 and -2), specificity protein 1 (SP1), NF-κB and polyoma enhancer activator 3/E26 (St-Pierre et al., 2004). Through the Ras/Raf/MEK/ERK pathway, these transcriptional factors are activated and translocated into the nucleus to regulate MMP9 gene expression (Chang et al., 2011; Garavello et al., 2010; Wu et al., 2013). The correlation between ERK1/2 and MMP9 is quite controversial given that their regulation can be in opposition in different cell types. As upregulation of MMP9 is associated with activation of MEK/ERK in neurological diseases such as ischemic stroke (Maddahi et al., 2009), we examined whether Erk1/2 also elevated Mmp9 in *fn40a* mutants. Western blot analysis showed that phosphorylation of Erk1/2 increased in the hemorrhagic mutants, and this was protected by miconazole treatment, compared with wild-type siblings treated with DMSO or miconazole at 24 hpf (Fig. 6H). Importantly, by comparing these results with those from heterozygous mutants in the absence or presence of miconazole (Fig. S8A′-B′;F′-G′), pErk in the brain vessels of the *fn40a* mutant increased at 36 hpf (Fig. S8C,C′) and at 48 hpf (Fig. S8H,H′), which decreased after miconazole treatment (Fig. S8D,D′,I,I′), suggesting that miconazole is able to suppress elevated pErk in the brain vessels of this mutant.

Furthermore, each of the three MEK inhibitors U0126, PD0325901 and SL-327 effectively suppressed the brain hemorrhage rate of *fn40a* mutants in a dose-dependent manner (Fig. 6I; Fig. S7B-D) (Shin et al., 2016). Although PD0325901 was somewhat toxic to embryos, its suppression was highly efficient. Remarkably, when a MEK inhibitor (U0126 or PD0325901) and miconazole were combined at lower doses they synergistically inhibited brain hemorrhages in *fn40a* mutants (Fig. 6J-K); each was less effective in suppressing hemorrhages when used alone. When assessing the hemorrhage rate of mutants treated with miconazole or MEK inhibitors (or combined as in Fig. 6I-K), we considered that mutants with little or no hemorrhage were ‘hemorrhage-negative’ and hemorrhagic mutants treated with DMSO were ‘hemorrhage-positive’. Western blot analysis confirmed that miconazole and U0126 (both at a lower concentration of 3 µmol/l) synergistically decreased the pErk/tErk ratio and accordingly decreased Mmp9 in this hemorrhagic mutant at 24 hpf (Fig. 6L). Taken together, our data suggest that miconazole suppresses brain hemorrhages in *fn40a* mutants by decreasing Mek/Erk-regulated Mmp9 activity to protect the integrity of brain vessels.

## DISCUSSION

Strokes can be split into ischemic and hemorrhagic types. Although hemorrhagic stroke has a higher mortality than ischemic stroke, with about double the incidence in Asians compared with other ethnic groups (van Asch et al., 2010), we have gained fewer mechanistic insights into hemorrhagic stroke and so limited therapeutic interventions are available for this disease. To identify the intervention targets for hemorrhagic stroke, investigators have identified several mutations in monogenetic stroke syndromes, including NOTCH3 in cerebral autosomal dominant subcortical infarcts and leukoencephalopathy, COL4A1 in COL4A1-related brain small-vessel disease, and KRIT1, CCM2 and PDCD10 in cerebral cavernous malformations (Li and Whitehead, 2010; Lindgren, 2014; Rost et al., 2008). Genome-wide association studies have identified several single nucleotide polymorphisms (SNPs) associated with sporadic hemorrhagic strokes, including *APOE* (19q13), *SLC25A44* (1q22), *COL4A1* (13q34) and *KCNK17* (6p21) (Lindgren, 2014; Rost et al., 2008). These genetic studies of stroke might eventually lead to the identification of therapeutic targets, although none of them has yet been translated into the clinic. Mutations of *Col4a1* in mice recapitulate the clinical spectrum of disease, including intracerebral hemorrhages, retinal vascular tortuosity and glomerular basement membrane defects (Gould et al., 2006). Other mouse/rat models of hemorrhagic stroke by performing blood injection or collagenase injection are also reported. The autologous blood injection model mimics intracerebral hematoma but represents nothing about the vascular ruptures causing this disease (MacLellan et al., 2008, 2010). The collagenase injection model mimics vascular rupture processes but does not mimic the tissue-specific proteolysis that requires extreme caution of manipulation (Han et al., 2011; Wang et al., 2003). While these mouse/rat models are widely used for studying the molecular and cellular mechanisms of hemorrhagic stroke, no targets from these models have yet been translated into therapeutics (Keep et al., 2012). Here, we report that a known mutant *fn40a*, with significant intracerebral hemorrhages, is a hypomorphic allele to *m292* (Chen et al., 2001; Liu et al., 2007) but *fn40a* is capable of surviving to adulthood, a major feature for producing 100% offspring from mutant crossings. Brain hemorrhages begin ∼40 hpf, but hematomas are almost completely absorbed after 4 dpf. Inflammatory cells are also recruited to the hemorrhagic loci in this mutant. Furthermore, *β-pix*, which genetically interacts with *ccm1* and *pak2a* (Buchner et al., 2007; Gore et al., 2008; Liu et al., 2007), is downregulated, while the hemorrhage-induced inflammatory mediator *mmp9* is upregulated, suggesting that this mutant recapitulates many aspects of hemorrhagic stroke in humans. As demonstrated in our work, this mutant is suitable for high-throughput chemical screens for hemorrhagic suppressors. Identified small-molecule suppressors can not only be used as chemical probes for addressing the molecular mechanisms underlying hemorrhagic stroke, but also present a novel opportunity for drug discovery in this devastating disease. Therefore, our work presents a valuable hemorrhagic stroke model that can be exploited for both mechanistic studies and drug discovery.

Based on this study and previous work (Murthy et al., 2012), we propose that decreased expression of *β-pix* in *fn40a* mutants (hypomorphic allele) decreases Rac1 function to activate Mek1/2, which then increases the level of phosphorylated Erk and Mmp9 transcription/translation, leading to abnormal vascular permeability and disruption of vascular integrity. As blood circulation is enhanced and blood pressure increases after 24 hpf, these fragile vessels are inclined to break down and bleed, leading to brain hemorrhages in this mutant. By performing a chemical suppressor screen, we found that miconazole suppressed the intracerebral hemorrhages in *fn40a* mutants. Miconazole is an approved antifungal agent with a fungus-specific binding site against ergosterol biosynthesis and is pharmacologically described as an inhibitor of sterol demethylase and cell wall synthesis (Pierard et al., 2012). We have demonstrated that miconazole suppresses brain hemorrhages and vascular permeability by downregulating pErk and Mmp9 in *fn40a* mutants. More importantly, by injecting uPA into rats, we established a mesenteric hemorrhage model that shares the same MEK/ERK/MMP9 pathway underlying vessel rupture and hemorrhage. As in zebrafish, miconazole rectified the overexpression of MMP9 and suppressed mesenteric hemorrhages in rats. Therefore, miconazole works through highly conserved target(s) to protect vascular integrity from zebrafish to mammals. Its direct target(s) remain to be identified.

Clinical therapies for hemorrhagic stroke have not yet progressed beyond the introduction of thrombin during the hematoma-forming process and neuron protection or blood-clot surgery after the formation of a hematoma (Keep et al., 2012). Generally, patients with hemorrhagic strokes have a high percentage of capillary bleeding, with tiny amounts of blood before a lethal hemorrhage occurs. This opens a window for prevention and/or intervention. In the clinic, patients with ischemic stroke administered thrombolytic agents such as tPA have a rather high probability of hemorrhage transients, and miconazole may protect patients from such transients. Based on work in zebrafish and rats, we propose that miconazole may be a good candidate for these types of prevention of hemorrhagic stroke. Future clinical trials and mechanistic studies need to bring this promising discovery from basic research into the clinic.

## MATERIALS AND METHODS

### Animals

Zebrafish (*Danio rerio*) were maintained and bred as described in the Zebrafish Book (Westerfield, 2000). The Tg(*fli1a*:nuc-eGFP) (Roman et al., 2002), Tg(*coro1a*:eGFP) (Li et al., 2012), Tg(*flk1*:eGFP) (Beis et al., 2005) and Tg(*gata1*:DsRed) (Traver et al., 2003) zebrafish were described previously. The *fn40a* mutant was isolated from a large-scale mutagenesis screen of the zebrafish genome at Massachusetts General Hospital, Boston (Chen et al., 2001). Wild-type Wistar rats (*Rattus norvegicus*, ∼200 g, males) were purchased from Vital River, Beijing. The animals were raised and handled in accordance with the animal protocol IMM-XiongJW-3 for this study, which was approved by the Institutional Animal Care and Use Committee of Peking University that is fully accredited by AAALAC International.

### Chemical screen in zebrafish

We raised homozygous *fn40a* mutant embryos to adults and selected those adult pairs that consistently produced almost 100% hemorrhagic mutant embryos for this chemical screen. This hemorrhagic suppressor screen consists of three major steps. We started to perform a primary screen with 96-well plates. After collecting, sorting and rinsing the embryos, we arrayed five fertilized and healthy embryos before 6 hpf in each well of the 96-well plates, which were filled with 200 µl E3 medium. Chemical compounds were dissolved in DMSO to form a stock concentration of 20 mmol/l, and the compounds were diluted with E3 medium to form a working concentration of 10 µmol/l in 96-well plates, and compounds were applied at 6 hpf. Treated embryos were incubated in an incubator at 28°C, and compounds were washed off and replaced with fresh E3 medium at 24 hpf. Each compound was repeated in 3 wells. Hemorrhage rate was calculated at 2 dpf and 3 dpf, as the percentage of the number of hemorrhagic mutants after compound treatment out of the total hemorrhagic mutants used. The morphological changes/abnormalities of mutant embryos were also documented. We set up a criterion to choose candidate compounds wherein the compounds were capable of decreasing the hemorrhage rate to <60% but caused minimal embryonic lethality. The primary screen led us to identifying 85 candidate hemorrhagic suppressors. Second, we verified each candidate compound for its efficacy with more than 200 embryos in a 10 cm dish. We arrayed about 70 mutant embryos for each dish and with three dishes for each compound. The *fn40a* mutant embryos were then treated with 10 µmol/l of each compound starting from 6 to 24 hpf, compounds were washed off at 24 hpf, and the hemorrhage rate/morphological abnormalities were documented at 2 dpf and 3 dpf. This second verification screen resulted in 21 candidate compounds. Third, we carried out the final screen on testing dose-dependent efficacy and toxicity of the 21 candidate compounds. For those compounds that caused some embryonic abnormalities, we set up the concentration gradients as 0 (DMSO control), 2 µmol/l, 4 µmol/l, 6 µmol/l, 8 µmol/l and 10 µmol/l. For those compounds that caused minimal embryonic abnormalities, we first set up a wider range of concentrations as 0 (DMSO control), 1 µmol/l, 5 µmol/l, 10 µmol/l, 20 µmol/l and 40 µmol/l, and then gradually narrowed down the range to the final concentration gradient from 0 (DMSO control), 1 µmol/l, 2 µmol/l, 3 µmol/l, 4 µmol/l to 5 µmol/l. The final candidate compounds were chosen based on their efficacy and minimal toxicity by using the narrowest concentration gradients. About 40 mutant embryos were used for each experimental condition/concentration group in 6-well plates and repeated three times. After the three-step screen, we identified seven candidate hemorrhagic suppressors and five other compounds that caused different embryonic phenotypes such as abnormal dorsal-ventral patterning and arrhythmia.

### Chemical library and small-molecule inhibitors

An in-house small-molecule library containing 923 chemicals from the Chinese National Compound Library Center, Beijing, China, as well as MMP9 inhibitor I (444278, Calbiochem), MMP9 inhibitor II (444293, Calbiochem), MMP9 inhibitor V (444285, Calbiochem), U0126 (U0120, Sigma-Aldrich), PD0325901 (PZ0162, Sigma-Aldrich), and SL-327 (S1066, Selleck) were applied to siblings or *fn40a* mutant zebrafish embryos. Imidazole members of sulconazole, tioconazole, voriconazole, fluconazole and ketoconazole were also supplied by the Chinese National Compound Library Center. Treated embryos were collected and embedded in 1% low-melting point agarose for taking live images, or fixed in 4% paraformaldehyde for *in situ* hybridization, *O*-dianisidine staining or fluorescence immunohistochemistry.

### MMP9 zymography assay

Homozygous *fn40a* mutants were treated with DMSO, MMP9 inhibitor I (3 µmol/l), MMP9 inhibitor II (3 µmol/l), or miconazole (3 µmol/l) from 6 to 24 hpf, and treated embryos at 24 hpf were collected for protein preparation with a non-reducing protein lysis buffer (C1050, Applygen) that contains phenylmethyl sulfonyl fluoride (Sigma-Aldrich). Protein concentrations were quantified by bicinchoninic acid (BCA) protein assay kit (P1511, Applygen). We used each protein sample (40 mg) for MMP9 zymography assay (P1700, Applygen) according to the manufacturer's protocol. Proteinase activity was visualized as a clear band on Coomassie blue-stained gels (SDS-PAGE with substrate G from Applygen), as previously reported (Zhang et al., 2013).

### *O*-dianisidine staining and Tg(*gata1*:DsRed) for labeling erythrocytes

Applying 500 μl fresh staining solution (2 ml of 14% *o*-dianisidine; 500 μl of 0.1 mol/l NaOAc, pH 4.5; 2 ml of deionized H_2_O; 100 μl of H_2_O_2_) to live, dechorionated embryos, keeping in dark for 20 min, and washing three times with deionized H_2_O as previously described (Wingert et al., 2004). Stained embryos were imaged. Alternatively, Tg(*flk1*:eGFP; *gata1*:DsRed) double transgenic zebrafish were bred with wild-type or *fn40a* mutant to generate *fn40a*^+/−^;Tg(*flk1*:eGFP; *gata1*:DsRed) and *fn40a*^−/−^; Tg(*flk1*:eGFP; *gata1*:DsRed). The embryos treated with DMSO or miconazole were embedded in 1% low-melting point agarose, and live images of brain regions at 2 dpf were taken with a Zeiss LSM700 confocal microscope. The regions of interest were restricted to the hindbrain, exactly posterior to the middle cerebral vein, and the same depth of *z*-stacks was used from all embryos. The Tg(*gata1*:DsRed) signals for labeling erythrocytes were quantified.

### Fluorescence immunohistochemistry

Zebrafish embryos and rat tissues underwent cryosection at 5 to 7 µm and collected on gelatin-covered slides. Sections were then processed for fluorescence immunohistochemistry. The primary antibodies were anti-MMP9 (HPA001238, lot D104322, Sigma-Aldrich, USA, 1:100) for rat tissues, anti-ZO1 (339100, lot 823765A, Invitrogen, USA, 1:100), anti-CD31 (DIA-310, Dianova, Germany, 1:50), anti-laminin (Lama1, L9393, lot 103M4779, Sigma-Aldrich, USA, 1:100), anti-collagen type IV (ab6586, lot GR193836-3, Abcam, UK, 1:100), anti-Mmp9 (55345, lot OG2101, Anaspec, 1:50) for fish embryos, and anti-phospho-p44/42 MAPK (Erk1/2) (4370, lot 15, Cell Signaling Technology, 1:100) for fish embryos. The secondary antibodies were Alexa Fluor 488 goat anti-rabbit IgG (A11008, lot 1705869, Invitrogen, USA, 1:200), Alexa Fluor 555 goat anti-mouse IgG (A21424, lot 1631208, Invitrogen, USA, 1:200), Alexa Fluor 555 goat anti-rat IgG (A21434, lot 1670155, Invitrogen, USA, 1:200), and Alexa Fluor 555 goat anti-rabbit IgG (A21428, lot 1608466, Invitrogen, 1:200).

### Whole-mount RNA *in situ* hybridization

Digoxigenin-labeled antisense RNA probes were synthesized by *in vitro* transcription according to a standard protocol, and the *mmp9* RNA probe was made as previously described (Hillegass et al., 2008). The *mmp9* probe fragment was amplified from zebrafish cDNA library with the forward primer (5′-GGGGATTTTGCCCTGATCGTGGA-3′) and a T7-containing reverse primer (5′-*TAATACGACTCACTATAGGG* TTCCAGTAGCGCCCGTCCTTGA-3′). T7 RNA polymerase was used to generate the antisense *mmp9* probe. Whole-mount *in situ* hybridization was performed as described in the Zebrafish Book (Westerfield, 2000).

### Microinjection of DAPI through the sinus venous

Basically, we used a protocol similar to that reported by the Watts group (Tam et al., 2012). Tg(*flk1*:eGFP) embryos at 1.5 and 2 dpf were anesthetized and injected with 12 nl of 0.85 mg/ml DAPI through the sinus venous. Embryos were incubated at 28°C for 30 min, and then imaged using a laser-scanning confocal microscope (LSM510, Carl Zeiss, Germany) at 488 nm and 405 nm.

### Western blot

Embryos (50 embryos at 2 dpf or 100 embryos at 24 hpf) were collected and rinsed. The yolk proteins were removed and 100 µl RIPA protein lysis was then applied for isolating proteins. Proteins were resolved on 10% or 12% SDS-PAGE gels and transferred onto polyvinylidene difluoride membrane (Bio-Rad). The primary antibodies were anti-Mmp9 (55345, lot OG2101, AnaSpect, USA, 1:500), anti-MMP9 (HPA001238, lot D104322, Sigma Aldrich, 1:1000), anti-phospho-p44/42 MAPK (Erk1/2) (4370, lot 15, Cell Signaling Technology, 1:2000), anti-p44/42 MAPK (Erk1/2) (4695, lot 14, Cell Signaling Technology, 1:2000), anti-α-tubulin (BE0031, Easybio, 1:2000) and anti-GAPDH (TA-08, Golden Bridge, 1:2000). The secondary antibodies were goat anti-rabbit IgG-HRP (M21001, Abmart, China, 1:5000) and goat anti-mouse IgG-HRP (M21002, Lot 264474, Abmart, 1:5000). The stained membranes were imaged using a Bio-Rad ChemiDoc MP Imaging System (USA) and the bands were analyzed using ImageJ.

### Quantitative real-time PCR

Total RNA from embryos at given stages was extracted using a TRIzol protocol as previously described (Talbot and Schier, 1999). For each sample, 50 embryos were collected at 2 dpf, and the experiments were independently repeated three times. Primers for *mmp2*, *mmp9*, *mmp13b*, *mmp14a*, *mmp14b* and *mmp23aa* are listed in Table S2 and were used to produce 100-150 bp fragments. Quantitative real-time PCR was carried out with SYBR Premix DimerEraser (RR091a, Takara, Japan).

### Transmission electron microscopy

As previously reported (Zhang et al., 2014), the embryos were harvested and fixed in 2% paraformaldehyde and 2.5% glutaraldehyde in 0.1 mol/l PBS at 4°C for overnight. After a brief rinse, embryos were post-fixed with 1% osmium tetroxide in 0.1 mol/l PBS for 2 h at room temperature, and then embedded in Epon 812. Ultrathin sections were stained with uranyl acetate and lead citrate, and stained sections were observed and photographed by transmission electron microscopy (JEM 1230; JEOL, Japan).

### Rat model for urokinase-induced mesenteric hemorrhage and intravital observation of mesenteric microcirculation

Rats were anesthetized by intramuscular injection of urethane (2 g/kg). Cannulae were inserted into the left internal jugular and femoral vein for administration of urokinase (30,000 IU/kg, Aladine, China) and miconazole (5 or 10 mg/kg) respectively. Miconazole or physiological saline (NS) was injected into animals half an hour before imaging the baseline. After the baseline was recorded, urokinase solution or NS was injected as quickly as possible. Rats were kept in 37°C incubator and mesenteric membranes were kept moist with warm NS. Images and records were documented at 30 min intervals for a total of 2 h. Animals were placed in the right-lateral decubitus position and the mesenteric microcirculation was observed under an inverted microscope (BX51WI, Olympus, Japan) equipped with a color monitor (TCL J2118A, TCL, China) and DVD recorder (DVR-R25, Malata, China). The hemorrhage spots and hemorrhagic area of each spot were calculated by Image-Pro Plus and ImageJ. The total spot number and the hemorrhagic area of the whole mesenteric membrane are presented for each time point with statistic curves.

### Statistical analyses

All experiments were repeated at least three times with more than 50 zebrafish embryos, and with five or more rats. Two-tailed Student's *t*-test, one-way ANOWA or two-way ANOVA with post test was performed to evaluate the significance of differences between experimental and control groups and data are expressed as means±s.e.m. *P<0.05 indicates a statistically significant effect.
